# The Pathogenic Yeast Candida parapsilosis Forms Pseudohyphae through Different Signaling Pathways Depending on the Available Carbon Source

**DOI:** 10.1128/msphere.00029-22

**Published:** 2022-05-09

**Authors:** Christopher B. Rupert, Laura N. Rusche

**Affiliations:** a Department of Biological Sciences, University at Buffalo, State University of New York, Buffalo, New York, USA; University of Georgia

**Keywords:** Candida, cyclic AMP, filamentation, glycerol, sirtuin

## Abstract

Candida parapsilosis is an emerging fungal pathogen that primarily affects immunocompromised patients in hospitals. A significant risk factor is the use of implanted medical devices, which support the growth of biofilms composed of a mixture of individual yeast cells and chains of elongated pseudohyphal cells. The morphological switch between these two forms is triggered by cues from the environment, including nutrient availability and temperature. We examined how different nutrient sources affect the balance between yeast and pseudohyphae and found that cells grown in the presence of five- or six-carbon sugars form more pseudohyphae at 30°C than at 37°C. Conversely, cells grown on glycerol, a three-carbon polyalcohol, form more pseudohyphae at 37°C. Furthermore, we found that different regulators influence pseudohyphal growth on glucose at 30°C compared with those on glycerol at 37°C. In particular, cAMP signaling and the sirtuin deacetylase Hst1 were required for pseudohyphal growth on glycerol at 37°C but not on glucose at 30°C. Finally, we found that the carbon source on which C. parapsilosis is grown can influence its ability to establish an infection in a wax moth model. Overall, this study reveals that environmental conditions affect not only the extent of pseudohyphal growth but also which pathways and regulators govern pseudohyphal formation.

**IMPORTANCE** Candida parapsilosis is one of the leading causes of hospital-acquired yeast infections and poses a significant risk to immunocompromised people. Two of its properties that contribute to infection are metabolic flexibility, to use a range of nutrients available in the host, and cellular dimorphism, to switch between round yeast cells and chains of elongated pseudohyphal cells. Uncovering the molecular mechanisms that regulate these processes could reveal new targets for antifungal drugs. We found that for C. parapsilosis, the balance between yeast and pseudohyphal cells depends on the nutrients available and the growth temperature. Moreover, these environmental changes can affect its ability to cause infections. Finally, we found that a potential sensor of the cell’s metabolic state, the sirtuin Hst1, contributes to pseudohyphal growth for cells grown on glycerol. These findings indicate that the shape and virulence of C. parapsilosis likely vary depending on its location in the host.

## INTRODUCTION

Immunocompromised people face a significant health threat in the form of hospital-acquired *Candida* infections, or candidiasis. Of the species that cause candidiasis, Candida albicans is the most common and best studied. However, several other species are observed frequently, including Candida parapsilosis, one of the five most prevalent species causing candidiasis (1–6). C. parapsilosis is also considered an emerging pathogen, as the number of infections is increasing (6). C. parapsilosis is primarily hospital acquired, with the main route of infection being through surgical implants or catheter lines into the bloodstream (7, 8). The highest risk groups for C. parapsilosis infection are premature infants and the elderly (9, 10). Despite its increasing prevalence, C. parapsilosis is understudied compared with C. albicans, making it crucial to investigate the biological processes that underlie infections.

Upon entering a human host, pathogenic yeasts encounter stresses, including increased temperature, oxidative bursts from immune cells, and poor nutrient environments (11–14). These changes in the environment must be sensed and overcome for infection to be established. The ability to utilize a range of different carbon sources is crucial for establishing and maintaining infection. These carbon sources can be sugars, such as the six-carbon glucose found in the bloodstream, or smaller two- or three-carbon molecules. For example, glycerol forms the backbone of phospholipids and triglycerides and can be elevated in the bloodstream of patients with metabolic diseases, such as diabetes (15, 16). In addition to being nutrient sources, carbon compounds can also serve as signaling molecules that trigger cellular responses appropriate for the nutrient environment (17).

One of the biological responses required for infection is an ability to switch between dispersed and filamentous forms. For C. parapsilosis, the dispersed form consists of round yeast cells that detach from one another after cell division, whereas the filamentous form consists of elongated cells that remain attached in chains called pseudohyphae (18, 19). Unlike C. albicans, C. parapsilosis does not form true hyphae, which are long germ tubes lacking distinct cell boundaries (20). Instead, C. parapsilosis forms pseudohyphae, which have visible constrictions between cells. This dimorphic switch between dispersed and filamentous cells is required for virulence in multiple pathogenic yeast species, with each cell type contributing differently to an infection (21, 22).

Switching from yeast cells to a filamentous form is triggered by environmental stimuli whose presence is transduced by signaling pathways. These pathways ultimately activate transcription factors that drive the expression of genes necessary for filamentation. One major pathway that controls filamentous growth in the well-studied C. albicans is the cyclic AMP (cAMP)/protein kinase A (PKA) pathway (23). In this pathway, the enzyme adenylate cyclase is activated by multiple upstream inputs, including elevated temperature, *N*-acetylglucosamine, serum, and carbon or nitrogen starvation (12, 21, 24, 25). Once activated, adenylate cyclase synthesizes cAMP from ATP. The cAMP then activates PKA, which subsequently phosphorylates effector proteins, including transcription factors that activate genes required for filamentous growth (23, 26, 27). One notable transcription factor activated by the cAMP/PKA pathway in C. albicans is Efg1, a master regulator of filamentous growth (28–30).

Much remains unknown about how *Candida* yeasts regulate signaling pathways that induce pathogenic responses, such as filamentation. One family of potential regulators is the sirtuin deacetylases, which remove acetyl groups from protein lysines. Sirtuins are distinct from other deacetylases in that the deacetylation reaction requires NAD^+^, which has led to the hypothesis that these deacetylases can sense changes in the nutrient environment that impact intracellular NAD^+^ levels (31). Target proteins of sirtuins include histones, which repress transcription when unacetylated, and other proteins whose function or interactions can be modified by acetylation (32, 33).

Sirtuins regulate virulence-associated traits in some pathogenic yeast. For example, in Candida glabrata, the sirtuins Sir2 and Hst1 repress genes involved in cell adhesion and oxidative stress response (14, 34). When this yeast is grown under conditions that lower intracellular [NAD^+^], such as in the urinary tract, sirtuin activity decreases, inducing the expression of genes required for infection. Additionally, we found recently that in C. albicans, the sirtuin Sir2 contributes to the formation of hyphae and expression of hyphal-specific genes (35). However, similar deacetylases in C. parapsilosis have not been investigated.

In this study, we investigated how different carbon sources affect pseudohyphal growth and virulence in C. parapsilosis, and we found that the impact of a particular carbon source varied depending on the growth temperature. We also found that pseudohyphal growth was dependent on adenylate cyclase activity and the sirtuin deacetylase Hst1 but only under certain growth conditions. Therefore, to develop effective treatments for C. parapsilosis, virulence traits, such as pseudohyphal growth, should be studied under conditions that mimic those found in a human host.

## RESULTS

### C. parapsilosis formed pseudohyphae when grown on sugars at 30°C or glycerol or acetate at 37°C.

**FIG 1:**
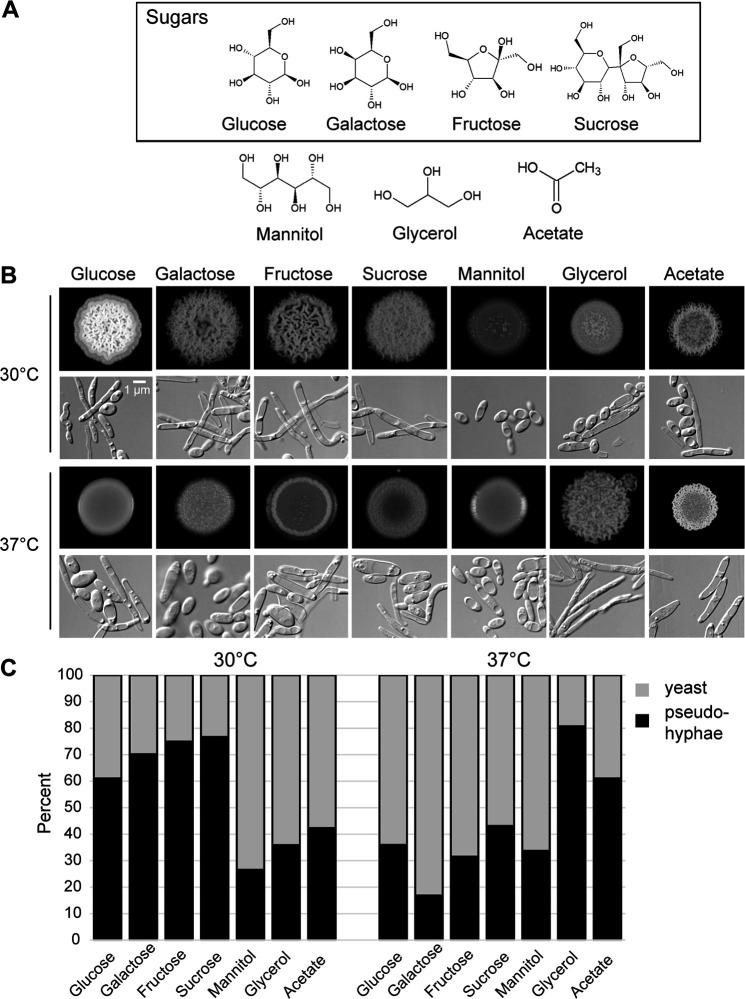
Carbon source and temperature influenced the growth pattern of C. parapsilosis. (A) Molecular structures of the carbon sources used in this study. (B) Colony and cell morphology of wild-type C. parapsilosis cells (CLIB214) grown on rich medium (yeast extract and peptone; YP) containing the indicated carbon sources at 30°C (top) and 37°C (bottom). Spots were inoculated with 10^5^ cells in 10 μL H_2_O. Spots and cells from the pictured spots were imaged after 3 days of growth and were representative of two separate experiments done in duplicate. (C) The proportions of yeast and pseudohyphae were determined for spots grown on each carbon source. Each bar represents the average of two separate experiments. For each one, approximately 150 cells were scored from 8 to 12 fields of view.

To determine how the available carbon source influences cell morphology, we grew the C. parapsilosis clinical isolate CLIB214 on agar medium containing various carbon compounds, including five- or six-carbon sugars, the polyols mannitol and glycerol, and the organic acid acetate (Fig. 1A). Cells were grown in spots seeded with approximately 10^5^ cells to generate communities that had morphologies similar to those described previously (19). Growth was either at 30°C, which is the standard temperature for laboratory yeast, or 37°C, which is human body temperature. On the standard carbon source glucose, C. parapsilosis communities adopted a crepe morphology composed mostly of pseudohyphae when grown at 30°C but adopted a smooth morphology composed mostly of yeast at 37°C (Fig. 1B, column 1). Similar morphologies were observed for the other sugars tested, namely, galactose, fructose, and sucrose (Fig. 1B, columns 2 to 4). However, the pattern was reversed for glycerol, with a smooth morphology composed mostly of yeast at 30°C and a crepe morphology composed of long, thin pseudohyphae at 37°C (column 6). Communities grown on mannitol were consistently smooth and composed of yeast cells (column 5), whereas those grown on acetate had a crepe morphology, which was enhanced at 37°C (column 7). To determine the percentage of yeast and pseudohyphal cells in each growth condition, we defined pseudohyphal cells as forming chains with box-shaped ends and a length-to-width ratio greater than three, whereas yeast cells had oval-shaped ends and lower length-to-width ratios. We found that the crepe communities were composed of 60% to 80% pseudohyphae, whereas the smooth communities consisted of 60% to 80% yeast (Fig. 1C). These observations reveal that both carbon source and temperature affect the balance between yeast and pseudohyphae in C. parapsilosis.

### Transcription factors associated with filamentous growth were not expressed similarly under two conditions that produce pseudohyphae.

**FIG 2:**
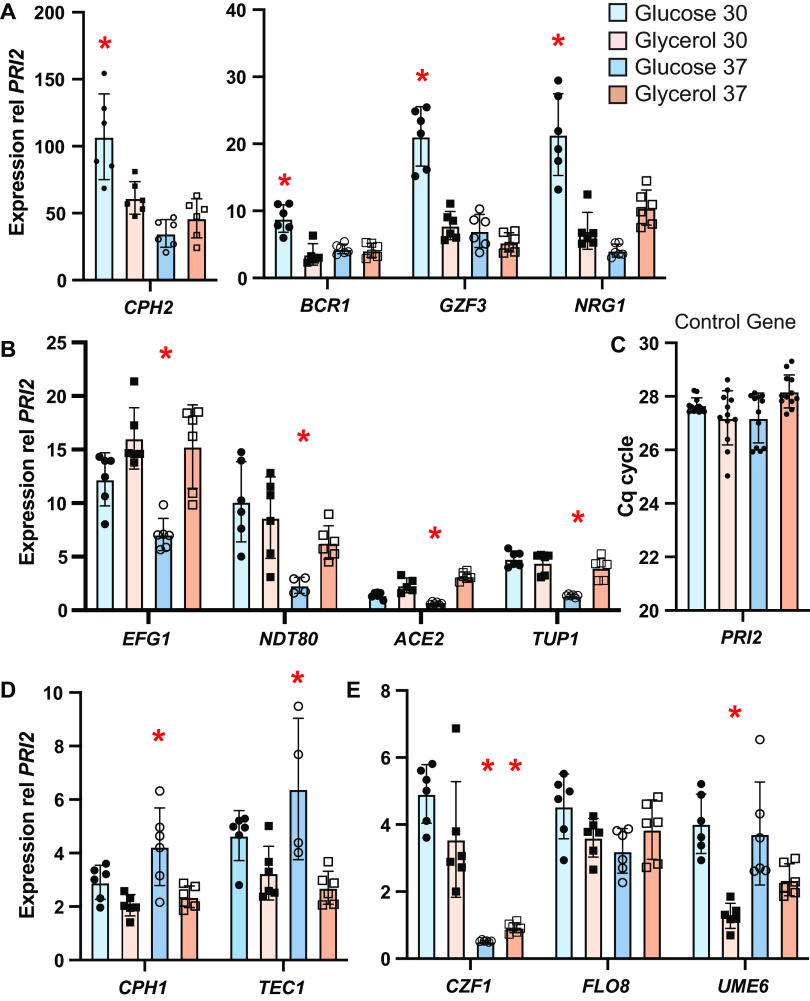
Transcription factor genes were expressed differently under the two filament-promoting conditions. RNA was extracted from wild-type CLIB214 cells grown on solid medium (YP) containing glucose (blue bars) or glycerol (orange bars) at 30°C (lighter bars) or 37°C (darker bars). Each bar represents the average and standard deviation of six independent extractions, with expression normalized to the control gene *PRI2*. Samples that were statistically different (*P < *0.05) based on two-way ANOVA with Tukey *post hoc* analysis are indicated by an asterisk (*). (C) For the control gene *PRI2*, the threshold cycle (*C_q_*) did not vary across conditions, indicating that *PRI2* is expressed consistently.

To elucidate the signaling pathways that drive filamentous growth, we next investigated which pseudohyphal-associated transcription factors are expressed under various growth conditions. For these experiments, we focused on two conditions that produce filamentous growth, namely, glucose at 30°C and glycerol at 37°C. In both C. parapsilosis and C. albicans, filamentous growth is triggered by a network of transcription factors that generate a feedforward loop by binding to their own promoters as well as those of other transcription factors (24, 29, 30, 36). Thus, activated transcription factors are likely to have a higher expression than inactive factors. We therefore measured the expression of candidate transcription factors using reverse transcriptase quantitative PCR (RT-qPCR). Interestingly, transcription factor genes were not expressed similarly under the two filamentous conditions. Moreover, transcription factors could be grouped by their expression patterns across the different temperatures and carbon sources. One group, namely, *CPH2*, *BCR1*, and *GZF3*, was expressed most highly on glucose at 30°C (Fig. 2A). A second group, *EFG1*, *NDT80*, *ACE2*, and the repressor *TUP1*, had the lowest expression on glucose at 37°C (Fig. 2B). *NRG1*, which associates with *TUP1* in a corepressor complex in C. albicans, shared features with both groups. Expression was highest on glucose at 30°C and lowest on glucose at 37°C (Fig. 2A). Finally, *CPH1* and *TEC1* were most expressed on glucose at 37°C (Fig. 2C). The other factors investigated, namely, *CZF1*, *FLO8*, and *UME6*, each had unique expression patterns (Fig. 2D). Thus, under the two conditions that result in pseudohyphal growth, different transcription factor genes were elevated, suggesting that distinct combinations of signaling pathways are active.

### cAMP was required for pseudohyphal growth on glycerol at 37°C.

**FIG 3:**
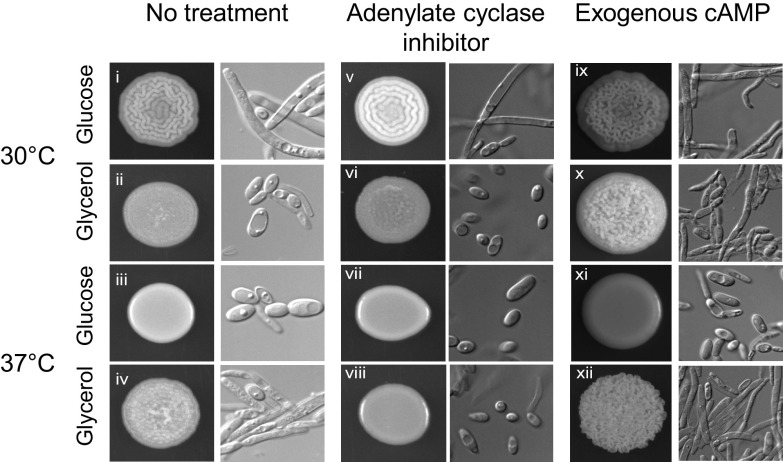
Pseudohyphal growth on glycerol at 37°C was dependent on adenylate cyclase. Wild-type cells (CLIB214) were spotted onto solid medium containing glucose or glycerol, with or without 10 mM cAMP or 50 μM MD-12,330A, an inhibitor of adenylate cyclase. Spots and cells were imaged after 3 days of growth. The experiment was performed in duplicate two separate times, with similar results.

One of the main signaling pathways that induces filamentous growth in C. albicans is the cAMP/PKA pathway, which phosphorylates the transcription factor Efg1 (37). To determine whether cAMP/PKA activation also promotes filamentous growth in C. parapsilosis, we grew cells in the presence of MDL-12,330A, an inhibitor of adenylate cyclase, which is a key component of the cAMP/PKA pathway. Indeed, on glycerol at 37°C, MDL-12,330A abolished the crepe community morphology and attenuated pseudohyphal growth (Fig. 3, compare iv and viii). However, on glucose at 30°C, the crepe community morphology and pseudohyphal growth persisted in the presence of the adenylate cyclase inhibitor (Fig. 3, compare i and v). These results suggest that cAMP signaling is critical for pseudohyphal growth on glycerol at 37°C but that other pathways trigger pseudohyphal growth on glucose at 30°C. Thus, pseudohyphal growth is induced by different signaling pathways depending on the growth conditions, which is a finding consistent with the different expression patterns of transcription factor genes.

If cAMP signaling induces pseudohyphal growth in C. parapsilosis, adding exogenous cAMP should activate the pathway and alter the morphology of cells grown under conditions that typically result in a smooth community composed of yeast cells (glycerol at 30°C and glucose at 37°C). Indeed, for cells grown on glycerol at 30°C, exogenous cAMP induced a crepe community morphology composed of more pseudohyphae than for cells grown with no exogenous cAMP (Fig. 3, compare ii and x). However, the addition of exogenous cAMP did not alter community or cell morphology on glucose at 37°C (Fig. 3, compare iii and xi). These results suggest that although exogenous cAMP does trigger pseudohyphal growth under some conditions, on glucose at 37°C, cells are blocked from forming pseudohyphae. This finding reinforces the notion that different pathways affect pseudohyphal growth depending on carbon source and temperature.

### The deacetylase Hst1 contributed to pseudohyphal growth on glycerol at 37°C.

**FIG 4:**
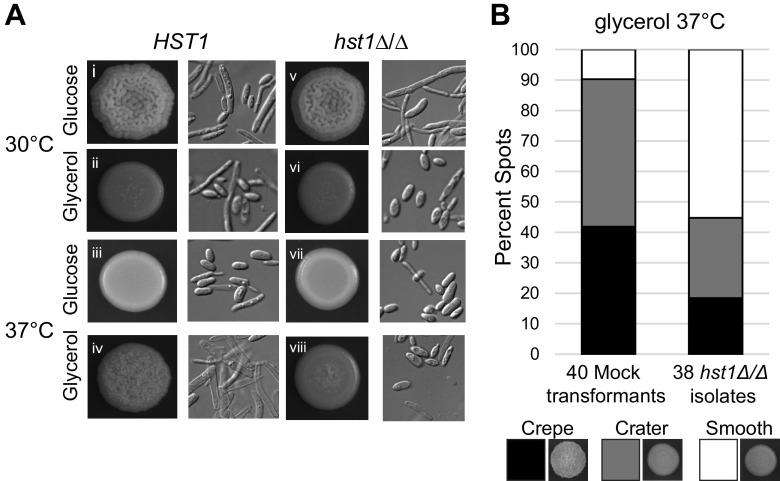
The deacetylase Hst1 was required for pseudohyphal growth on glycerol at 37°C. (A) Communities of wild-type (CLIB214) or *hst1Δ/Δ* (LRY3263) cells grown on glucose or glycerol. Spots and cells were imaged after 3 days of growth and were performed in duplicate in two separate experiments. Similar results were obtained for a second *hst1Δ/Δ* isolate (LRY3296). (B) Growth phenotypes on glycerol at 37°C for 38 independent *hst1Δ/Δ* isolates (LRY3259 to 3296) and 40 mock-transformed isolates (LRY3297 to 3331) exposed to a CRISPR plasmid lacking the *HST1*-directed guide RNA. Phenotypes were classified as crepe, crater, or smooth following published categories (19) and were scored in a blind manner by three observers evaluating two separate rounds of growth.

**FIG 5:**
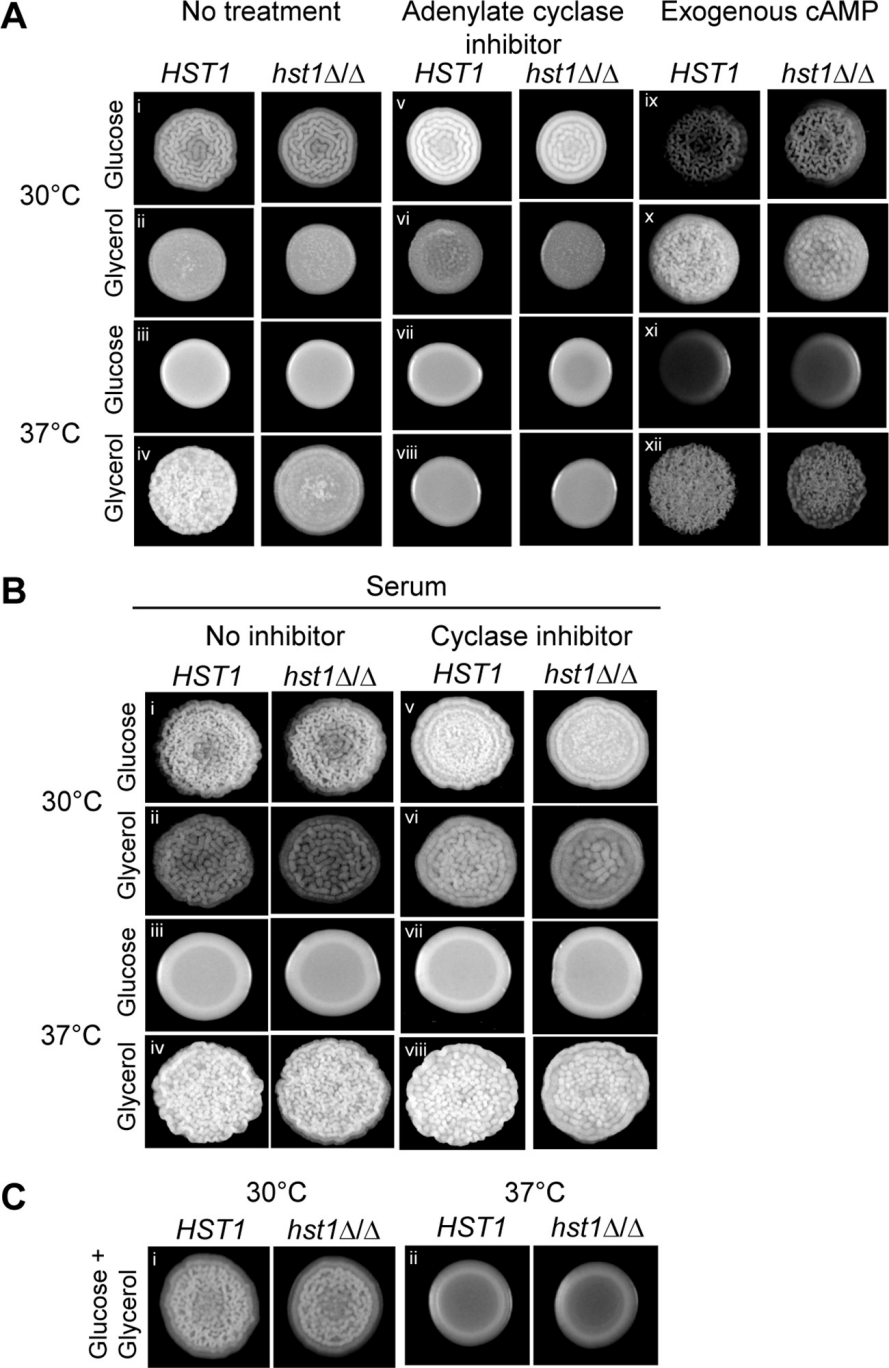
The pseudohyphal defect in *hst1Δ/Δ* cells could be rescued by cAMP or serum. (A) Wild-type (CLIB214) and *hst1Δ/Δ* cells (LRY3263) were spotted onto plates containing glucose or glycerol, with or without 50 μM MDL-12,330A or 10 mM cAMP. (B) Wild-type and *hst1Δ/Δ* cells were spotted onto plates containing glucose or glycerol with 10% fetal bovine serum and with or without MDL-12,330A. (C) Cells were spotted onto plates containing both glucose and glycerol. All spot pictures were taken after 3 days of growth and were performed in duplicate on two separate days. Similar results were obtained for another *hst1Δ/Δ* isolate (LRY3296).

**FIG 6:**
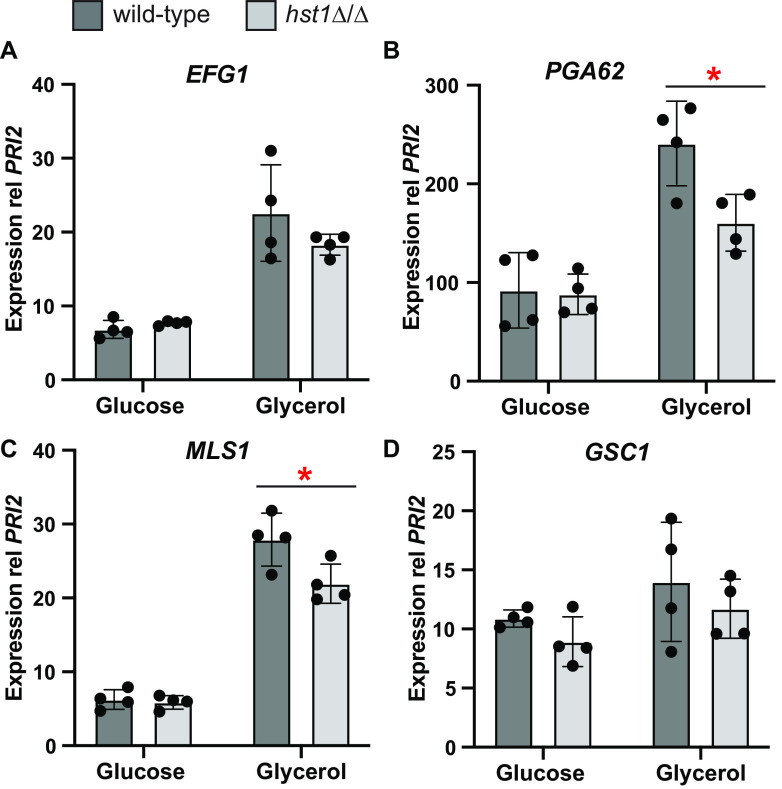
On glycerol at 37°C, *hst1Δ/Δ* cells had a lower expression of *EFG1* and Efg1-target genes than wild-type cells. RNA was extracted from wild-type (CLIB214) or *hst1Δ/Δ* (LRY3263 and 3296) spots grown 3 days on glucose or glycerol at 37°C. mRNAs were quantified by RT-PCR and normalized to the *PRI2* gene. Expression for the wild type represents four wild type spots harvested on two separate days, whereas expression for *hst1Δ/Δ* cells represents two spots each of LRY3263 and LRY3296 harvested on 2 days. A significant difference between wild-type and *hst1Δ/Δ* cells grown on glycerol was evaluated using Student’s *t*-test; *, *P < *0.05.

**FIG 7:**
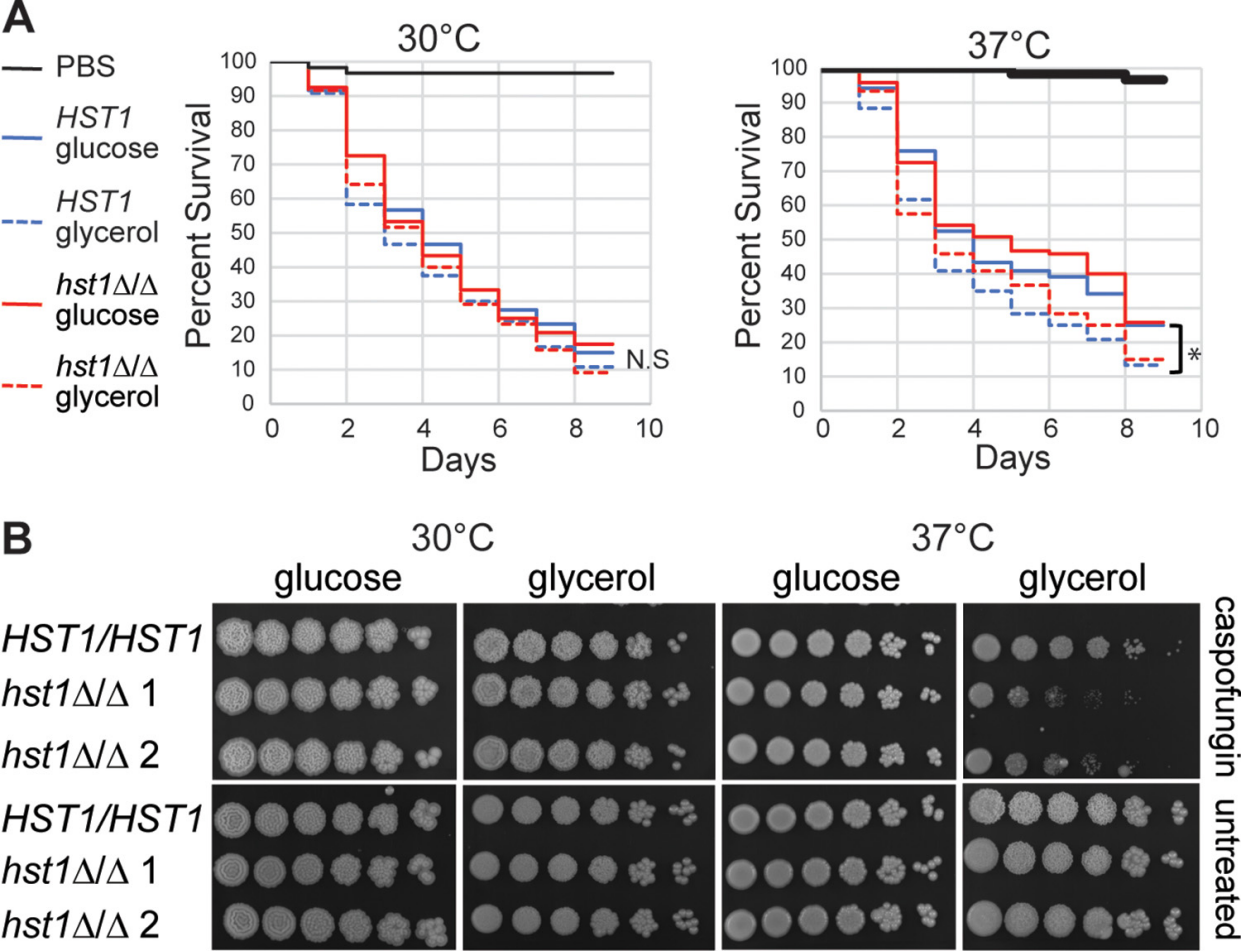
Effect of growth condition and *hst1Δ/Δ* mutation on virulence traits of C. parapsilosis. (A) Virulence assay using G. mellonella larvae. Wild-type (CLIB214) and *hst1Δ/Δ* cells (LRY3263 and LRY3296) were grown on solid medium containing glucose or glycerol at 30°C or 37°C for 3 days. Approximately 4 × 10^6^ cells were injected into each of 30 larvae, which were incubated at 30°C (left) or 37°C (right) for 8 days. Live larvae were counted every day. Lines represent the percent survival of 120 larvae from four different experiments. *, *P < *0.05; using log rank test. (B) Caspofungin sensitivity assay. Wild-type or *hst1Δ/Δ* cells (LRY3263 and 3296) were grown overnight in YPD at 30°C and then subcultured to an OD_600_ of 0.2 and grown until an OD_600_ of 2.5. Five-fold serial dilutions were spotted on plates with or without 0.5 μg/mL caspofungin and imaged after 2 days of growth.

Our lab reported recently that the sirtuin deacetylase Sir2 modulates filamentous growth in C. albicans (35). To determine whether sirtuins also influence filamentous growth in C. parapsilosis, we investigated the deacetylase Hst1. CpHst1 is an ancient paralog of C. albicans Sir2 and is orthologous to the sirtuins that contribute to virulence in C. glabrata (38). We focused on *CpHST1* because the ortholog of *CaSIR2* was lost in the C. parapsilosis lineage (38). We created multiple *hst1Δ/Δ* deletion strains using a CRISPR system developed for C. parapsilosis (39). On glycerol at 37°C, these *hst1Δ/Δ* strains had a smooth community morphology and displayed fewer pseudohyphae than the parent strain (Fig. 4A), indicating that Hst1 promotes pseudohyphal growth. However, for other growth conditions, there were no differences in morphology between *hst1Δ/Δ* and parental cells (Fig. 4A). Although we were unable to reintroduce *HST1* for complementation analysis, we did create 38 independent *hst1Δ/Δ* isolates to determine the reproducibility of the observed phenotypes. To control for off-target effects, mutants were generated using three single guide RNAs (sgRNAs) targeting different regions of the *HST1* gene. Most *hst1Δ/Δ* strains had a smooth or crater morphology on glycerol at 37°C, whereas mock transformants were not (Fig. 4B). Therefore, Hst1 contributes to pseudohyphal growth of C. parapsilosis under particular conditions, such as on glycerol at 37°C. Further analysis of the *hst1Δ/Δ* phenotype (Fig. 5-7) was performed using two isolates generated with different sgRNAs. Both isolates consistently displayed a lack of pseudohyphal growth on glycerol at 37°C.

### The pseudohyphal defect of *hst1Δ/Δ* cells was overcome by exogenous cAMP or serum.

On glycerol at 37°C, pseudohyphal growth was lost when adenylate cyclase was inhibited (Fig. 3) and in the absence of the NAD^+^-dependent deacetylase Hst1 (Fig. 4A). To determine whether Hst1 acts upstream or downstream of adenylate cyclase, we investigated whether the loss of Hst1 could be overcome by adding exogenous cAMP. If the pseudohyphal defect in *hst1Δ/Δ* strains is due to the reduced activity of adenylate cyclase, adding cAMP should trigger pseudohyphal growth. However, if the defect is due to an inability to respond to cAMP, exogenous cAMP will not alter the cell morphology. We found that the addition of cAMP to cells grown on glycerol at 37°C induced the crepe community morphology and increased pseudohyphal growth of *hst1Δ/Δ* cells (Fig. 5A, compare *hst1Δ/Δ* cells in iv and xii). This finding suggests that Hst1 acts upstream of adenylate cyclase.

The observation that a loss of Hst1 affects pseudohyphal growth on glycerol at 37°C but not on glucose at 30°C (Fig. 4A) suggests that it acts only upstream of particular signaling pathways. To test this idea further, we determined whether *hst1Δ/Δ* cells were defective in pseudohyphal induction under another condition not expected to rely on cAMP/PKA signaling, namely, the addition of serum. In C. albicans, serum induces filamentous growth through both cAMP/PKA and mitogen-activated protein kinase (MAPK) pathways (21). We found that on glycerol at 37°C, 10% serum induced *hst1Δ/Δ* cells to form crepe community morphologies (Fig. 5B, iv), indicating the *hst1Δ/Δ* defect is overcome by the presence of serum. Importantly, the adenylate cyclase inhibitor MDL-12,330A did not inhibit pseudohyphal growth in the presence of serum (Fig. 5B, compare left and right pairs of columns), suggesting that pseudohyphal growth in the presence of serum does not depend on the cAMP/PKA pathway. Thus, the *hst1Δ/Δ* pseudohyphal defect was associated with conditions that rely on adenylate cyclase to trigger filamentation.

### Loss of *HST1* lowered expression of *EFG1* and pseudohyphal-associated genes on glycerol at 37°C.

The apparent reduction of cAMP/PKA signaling in *hst1Δ/Δ* strains grown on glycerol at 37°C could result in lower activity of the transcription factor Efg1, which is phosphorylated by PKA in C. albicans (40, 41). In turn, reduced Efg1 activity could lower the expression of pseudohyphal-specific genes as well as *EFG1* itself, of which all are expected to be targets of Efg1 (29). Therefore, we compared the expression in wild-type and *hst1Δ/Δ* cells of *EFG1* and three genes known to be directly regulated by Efg1, namely, *GSC1*, *PGA62*, and *MLS1* (29). On average, *hst1Δ/Δ* cells grown on glycerol at 37°C expressed less *EFG1* than wild-type cells, although the difference was not statistically significant (Fig. 6, top left). However, the Efg1-regulated pseudohyphal genes *PGA62* and *MLS1* were significantly reduced in *hst1Δ/Δ* relative to wild-type cells. In contrast, on glucose at 37°C, there was no significant difference in the expression of these genes between *hst1Δ/Δ* and wild-type cells. Therefore, in the absence of Hst1, Efg1 activation on glycerol appears to be reduced, which in turn influences the expression of pseudohyphal genes.

### Cells grown on glucose at 37°C were refractory to filamentous growth.

It was notable that on glucose at 37°C neither serum (Fig. 5B, iii) nor cAMP (Fig. 5A, xi) induced filamentous growth, although both compounds triggered filamentous growth on glycerol at 30°C and in *hst1Δ/Δ* cells (Fig. 5A, x and xii; Fig. 5B, ii and iv). These observations suggest that cells grown on glucose at 37°C are refractory to filamentation. To test whether the presence of glucose represses pseudohyphal growth, we grew cells on a combination of glucose and glycerol. We found that under these conditions, cells did not display filamentous growth at 37°C (Fig. 5C), even though glycerol triggers filamentation in the absence of glucose. This finding extends the model that different pathways affect pseudohyphal growth depending on the carbon source and temperature.

### Cells pregrown on glucose at 37°C were less virulent than cells pregrown under other conditions.

Filamentous growth is an important virulence trait, so we hypothesized that cells pregrown on carbon sources that induce pseudohyphal growth would “prime” cells to better infect a host. To test this idea, we injected Galleria mellonella larvae (42) with cells pregrown on solid medium containing glucose or glycerol at 30°C or 37°C. Injected larvae were maintained at the same temperature as the pregrowth cells. At 37°C, there was a statistically significant difference in survival (log rank test, *P < *0.05) between larvae infected with glucose-grown cells and glycerol-grown cells (Fig. 7A, right). This finding indicates that the carbon source available prior to infection had an impact on the ability of C. parapsilosis to establish infection. However, virulence in this assay did not correlate with pseudohyphal growth, as there was no difference in the virulence of cells grown on glucose or glycerol at 30°C (Fig. 7A, left). Moreover, there was no difference in virulence between wild-type and *hst1Δ/Δ* cells, regardless of carbon source or incubation temperature (Fig. 7A, both graphs). Thus, although the type of carbon source available prior to infection can affect the virulence of C. parapsilosis, its effect is probably not due its influence on pseudohyphal formation.

### Caspofungin sensitivity was increased in *hst1Δ/Δ* cells grown on glycerol at 37°C.

Another clinically relevant trait of fungal pathogens is their susceptibility to antifungal drugs. Moreover, the emergence of resistance to these drugs makes it important to identify new antifungal targets or mechanisms to increase the efficacy of existing agents. We therefore tested the sensitivity of wild-type and *hst1Δ/Δ* cells to the antifungal drug caspofungin. Caspofungin is used commonly to treat candidiasis and belongs to the echinocandin class of compounds, which block the synthesis of the cell wall polymer β-glucan by inhibiting β-1,3-glucan synthase (Gsc1 in C. parapsilosis). We compared the growth of wild-type and *hst1Δ/Δ* cells in the presence of a sublethal concentration of caspofungin (0.5 μg/mL). Although this concentration of caspofungin did not impair the growth of wild-type cells, it inhibited the growth of *hst1Δ/Δ* cells specifically on glycerol at 37°C (Fig. 7B, top right). No growth defects were observed for *hst1Δ/Δ* cells grown in the absence of caspofungin (Fig. 7B, bottom images). These results indicate that Hst1 regulates cellular processes that determine the sensitivity to caspofungin.

## DISCUSSION

**FIG 8:**
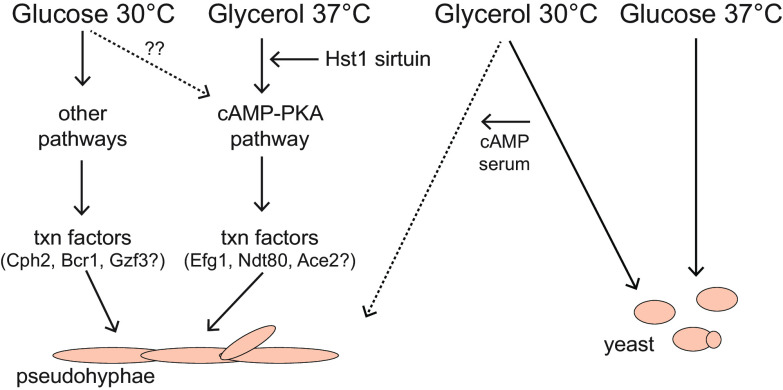
Model depicting pathways contributing to pseudohyphal growth depending on the available carbon source and the incubation temperature.

In this study, we found that, for C. parapsilosis, the available carbon source and temperature impact the balance between yeast and pseudohyphal morphologies. We propose a model in which different signaling pathways and transcription factors contribute to pseudohyphal growth depending on the carbon source and temperature (Fig. 8). In particular, we found that the cAMP/PKA pathway was required for pseudohyphal growth on glycerol at 37°C but not on glucose at 30°C, indicating that additional pathways can activate this growth pattern. We also found that the transcription factors Bcr1, Cph2, and Gzf3 may work together to regulate pseudohyphal growth on glucose at 30°C and that the transcription factors Efg1, Ndt80, and AceII may be important on glycerol at 37°C. Finally, the sirtuin deacetylase Hst1 acted upstream of adenylate cyclase to influence pseudohyphal growth and *EFG1* expression on glycerol at 37°C, revealing a role for sirtuins in regulating filamentation.

One caveat is that these experiments were performed using a single clinical isolate, CLIB214, and it is well documented that traits vary among isolates of C. parapsilosis (43–45). Nevertheless, we expect that our overall finding, that carbon source and temperature affect the extent of pseudohyphal growth, will hold true for other C. parapsilosis isolates. Although the specific responses may vary, it would be surprising if these environmental factors affect only the CLIB214 isolate.

### Signaling pathways that promote pseudohyphal growth.

We found that the cAMP/PKA pathway was required for pseudohyphal growth on glycerol at 37°C but not on glucose at 30°C (Fig. 3 and 8). This observation indicates that on glucose at 30°C, the cAMP/PKA pathway is either inactive or its absence can be compensated by other signaling pathways. Two pathways that could be active on glucose at 30°C are the Cek1/MAPK and RIM pathways. In C. albicans, the Cek1/MAPK pathway responds to inputs, such as temperature and nutrient stress, and triggers cell wall remodeling and invasive growth, and the RIM pathway senses pH and activates filamentous growth through proteolytic cleavage of Rim101 (46). Our observations also indicate that pseudohyphal growth may be inhibited more easily under specific conditions, such as glycerol at 37°C, for which it only takes the inactivation of cAMP/PKA to abolish this growth pattern. Understanding how C. parapsilosis behaves in different niches within the body will be key for developing effective antifungal treatments.

We noticed that cells grown on glucose at 37°C could not be induced to form pseudohyphae by either cAMP or serum, although these additives did induce pseudohyphae under other growth conditions (Fig. 5A and B). Moreover, growth at 37°C on medium containing both glucose and glycerol failed to induce pseudohyphal growth (Fig. 5C). These results suggest that at 37°C, the presence of glucose represses pseudohyphal growth. Related to these observations, we found that the virulence of C. parapsilosis was reduced when cells were pregrown on glucose at 37°C compared with that of the other three tested growth conditions (Fig. 7A). Thus, cells grown on glucose at 37°C behave differently than cells grown under other conditions, and it will be interesting to learn the basis for this difference. As an aside, the reduced virulence of cells grown on glucose at 37°C is probably not due to the lack of pseudohyphal growth, as other samples deficient in pseudohyphal growth, such as the *hst1Δ/Δ* strains and those grown on glycerol at 30°C, were not less virulent.

### Transcription factors required for pseudohyphal growth.

Our results suggest that the set of transcription factors that drive filamentation differ depending on the growth condition. The genes encoding Bcr1, Cph2, and Gzf3 were more highly expressed on glucose at 30°C than under other conditions (Fig. 2), suggesting that these transcription factors are active under this growth condition. All three of these factors contribute to pseudohyphal growth and biofilm formation in C. parapsilosis (47), and Bcr1 and Cph2 are reported to have overlapping functions based on similar expression profiles in *bcr1* and *cph2* deletion strains (47). Bcr1, Cph2, and Gzf3 are likely to be activated by a pathway other than cAMP/PKA, such as the MAPK or RIM signaling pathways, given that pseudohyphal growth on glucose at 30°C was unaffected by the inhibition of adenylate cyclase (Fig. 3).

For the other filament-promoting condition that we examined, glycerol at 37°C, there were no transcription factors whose expression was highest under this condition (Fig. 2). However, Efg1 was implicated as being important because the cAMP/PKA pathway was required for pseudohyphal growth (Fig. 3), and this pathway phosphorylates and activates Efg1 in C. albicans. Moreover, deletion of *HST1* reduced both filamentation and the expression of *EFG1* on glycerol at 37°C (Fig. 4 and 6). In C. parapsilosis, Efg1 target genes have been identified for cells grown in liquid yeast extract-peptone-dextrose (YPD; rich medium with glucose) at 30°C (29). We found that two Efg1 targets had higher expression on glycerol than that on glucose at 37°C (Fig. 6). Thus, Efg1 may be active on glycerol at 37°C.

*EFG1* had a similar expression pattern to two other transcription factors, namely, *NDT80* and *ACE2* (Fig. 2B), suggesting that they also play a role in pseudohyphal growth on glycerol at 37°C. Consistent with this idea, 70% of Efg1-regulated promoters also have a Ndt80 binding motif (29), suggesting that CpEfg1 and CpNdt80 regulate the same set of genes. Similarly, in C. albicans, there is significant overlap between genes directly regulated by CaEfg1 and CaNdt80 (30). Surprisingly, in C. parapsilosis, Efg1 and Ndt80 have opposite effects on filamentous growth and biofilm formation, with Efg1 promoting and Ndt80 repressing this behavior (48). It is therefore possible that Ndt80 and Efg1 tune the extent of pseudohyphal growth, such that increased activity of one factor relative to the other could shift the population toward yeast or pseudohyphal forms. Interestingly, CpEfg1 regulates *NDT80*, and there is a binding site for Ndt80 at the *EFG1* promoter (29). Thus, a feedback loop may balance the expression of these two factors.

### Contribution of deacetylase Hst1 to pseudohyphal growth.

We also uncovered a role for the sirtuin deacetylase Hst1 in promoting pseudohyphal growth on glycerol at 37°C but not on glucose at 30°C (Fig. 4A). The *hst1Δ/Δ* pseudohyphal defect could be overcome by the addition of exogenous cAMP (Fig. 5A) and correlated with a lower expression of *EFG1* and Efg1-regulated genes (Fig. 6). Therefore, Hst1 likely contributes to the activation of Efg1 through the cAMP/PKA pathway, acting upstream of adenylate cyclase. To understand its mechanism of action, it will be important to identify the deacetylation targets of Hst1. CpHst1 is orthologous to Hst1 and Sir2 in Saccharomyces cerevisiae, which generate repressive chromatin at subtelomeres, silenced mating-type loci, specific genes, and the rDNA repeats (49–52). C. parapsilosis lacks silenced mating-type loci, and we found that CpHst1 does not deacetylate histones at the rDNA or subtelomeres (38). Moreover, we did not detect promoter-specific binding of Hst1 through chromatin immunoprecipitation (ChIP) assays (data not shown). Therefore, Hst1 may deacetylate nonhistone targets. For example, it could deacetylate signaling proteins, such as receptors or adenylate cyclase, or it could influence signaling indirectly by regulating the expression of signaling proteins. In either case, Hst1 may allow cells to include information about intracellular NAD^+^ levels in determining cell morphology. Ample NAD^+^ would enable deacetylation by Hst1 and favor pseudohyphal growth, whereas low NAD^+^ would inhibit Hst1 and pseudohyphal growth.

We observed that *hst1Δ/Δ* cells were sensitive to the antifungal drug caspofungin when grown on glycerol at 37°C (Fig. 7B). Although it is preliminary, this finding suggests that combining a sirtuin inhibitor with caspofungin could be a useful treatment strategy. Although the caspofungin sensitivity and pseudohyphal growth defect of *hst1Δ/Δ* cells were both observed on glycerol at 37°C, it remains to be determined whether deacetylation of the same proteins affects both phenotypes.

### Summary.

In conclusion, we have determined that the signaling and transcriptional networks that activate pseudohyphal growth in C. parapsilosis vary depending on the environment, underscoring the importance of elucidating the environmental conditions experienced by C. parapsilosis at different sites of infection within the host. In addition, our work suggests that Hst1 or its deacetylation targets may be suitable candidates for new antifungal drugs.

## MATERIALS AND METHODS

### Yeast strains and growth conditions.

Candida parapsilosis strains used in this study (Table 1) were grown on medium containing 1% yeast extract, 2% peptone, and 2% of the specified carbon source (galactose, fructose, sucrose, mannitol, acetate, or glycerol), with solid medium also containing 2% Bacto agar. The adenylate cyclase inhibitor MDL-12,330A (Millipore Sigma; M182) was dissolved in dimethyl sulfoxide (DMSO) and used at 50 μM, cAMP (Fisher; AAJ6093606) was dissolved in sterile water and used at 10 mM, caspofungin (Sigma; SML0425-25MG) was dissolved in DMSO and used at 0.5 μg/mL, and serum (Gibco; 10437077) was added to a concentration of 10% (vol/vol). When selecting for transformants, cells were grown on YPD medium supplemented with 200 μg/mL nourseothricin (Goldbio; N-500-100).

**TABLE 1:** Strains used in this study

Strain	Genotype	Guide plasmid	Reference or source
CLIB214	Prototrophic	N/A^*a*^	19
LRY3259–3264	*hst1Δ/Δ*	pLR1306	This study (Fig. 4)
LRY3265–3280	*hst1Δ/Δ*	pLR1307	This study (Fig. 4)
LRY3281–3296	*hst1Δ/Δ*	pLR1308	This study (Fig. 4)
LRY3297–3331	Mock transformed	pCP-tRNA	This study (Fig. 4)
LRY3263	*hst1Δ/Δ*	pLR1306	This study (Fig. 5, 6 and 7)
LRY3296	*hst1Δ/Δ*	pLR1308	This study (Fig. 5, 6 and 7)

a N/A, not applicable.

CLIB214 is a prototrophic clinical isolate (19) of C. parapsilosis. Mutants lacking *HST1* were created using the pCP-tRNA Cas9 plasmid (39). Guide RNAs directed against *HST1* were designed using EupathGDT (http://grna.ctegd.uga.edu/), and three sequences with a composite quality score over 0.4 were selected (Table 2). These sequences were incorporated into the plasmid by annealing complementary oligonucleotides that had overhanging single strands compatible with the SapI cleavage sites in plasmid pCP-tRNA. The annealed oligonucleotides were ligated into the plasmid, and candidate plasmids were evaluated by diagnostic PCR and sequencing. A repair template to delete *HST1* was designed using 2 oligonucleotides (5′-ATAATTAAAGCGAGTTTAAGGAATTGTTTTGGTTACAGTTTTGAGCAGGAATCTGCATCACAATAGCGCC GTTCGAGGTT-3′ and 5′-CCCTTCATCTGCAACCTGCCATGTGGTATCACCGGTAACCTGCTATGTGACTTCACTCGCAACCTCGAAC GGCGCTATTG-3′) with 70 nucleotides corresponding to the sequences upstream or downstream of the *HST1* gene. The last 10 nucleotides of each primer annealed to the flanking sequence of the other primer, creating 20 bp of overlap between the 2 primers but not adding any additional sequence. These primers were extended by PCR to create a double-stranded repair template.

**TABLE 2:** Plasmids used in this study^*a*^

Name	Description	Sequence of:		Reference or source
		Forward guide RNA primer	Reverse guide RNA primer	
pLR1305	pCP-tRNA	N/A	N/A	39
pLR1306	HST1 guide 1	ccaTGAGAATGAAGGTGAGGACG	aacCGTCCTCACCTTCATTCTCA	This study
pLR1307	HST1 guide 2	ccaAAGCTACGCTGGAATACGACCGG	aacCCGGTCGTATTCCAGCGTAGCTT	This study
pLR1308	HST1 guide 3	ccAGTAAGGCATGCGACCGCAAGCGG	aaCCGCTTGCGGTCGCATGCCTTAC	This study

a Lower case letter represent nucleotides that remain single-stranded after annealing.

Transformations were carried out as described previously with some modifications (39). To delete *HST1*, CLIB214 cells were grown overnight in YPD at 30°C, then diluted to an optical density at 600 nm (OD_600_) of 0.2, and grown to mid-log phase at 30°C for 5.5 h (OD_600_, approximately 2.5). An equivalent of 50 OD of cells were spun down and washed once in 25 mL ice cold sterile water and then transferred to a 1.5-mL microcentrifuge tube in 1 mL of TEL (10 mM Tris, 1 mM EDTA, and 0.1 M lithium acetate). Cells were collected and resuspended in 500 μL TEL. A total of 100 μL of cells was added to transformation reaction mixtures containing 5 μg pCP-tRNA with the guide RNA sequence, 5 μg repair template, and 10 μg sheared salmon sperm DNA. Mock transformations contained 5 μg of pCP-tRNA with no guide RNA sequence and were used as a positive control for transformation and to create the mock transformants in Fig. 4. Cells were incubated at 30°C for 30 min and then 700 μL of PLATE (0.1 M lithium acetate, 10 mM Tris [pH 8.0], 1 mM EDTA, and 40% polyethylene glycol [PEG]) was added. After overnight incubation at 30°C, cells were heat shocked for 15 min at 44°C. Cells were collected and then resuspended in 200 μL YPD and allowed to recover for 4 h at 30°C with rotation. Colonies were screened by PCR, followed by RT-qPCR checking for *HST1* RNA.

Knockout strains were generated in five separate transformations. Eight positive colonies were selected from each transformation, except from the first, which yielded six knockouts. The first transformation was performed with pLR1306, the second and third with pLR1307, and the fourth and fifth with pLR1308 (Table 2). All transformations used the same repair template.

### Community morphology assays.

For spot morphology assays, cells were grown overnight in liquid YPD at 30°C, and then 1.0 OD equivalents of cells (approximately 10^7^ cells) was harvested and resuspended in 1 mL sterile water. A total of 10 μL of cells was spotted onto solid medium containing various carbon compounds and incubated for 3 days at 30°C or 37°C. Images were taken using a Bio-Rad ChemiDoc XRS+ imager under white light. Spot morphologies were scored using the crepe, crater, and smooth categories described previously (19). To reduce bias when *hst1Δ/Δ* and mock transformants were compared, spots were scored by three people with no knowledge of spot genotypes.

### Microscopy.

To visualize cell morphologies, an entire spot was harvested after 3 days of growth and suspended in phosphate-buffered saline (PBS; 137 mM NaCl, 2.7 mM KCl, 10 mM Na_2_HPO_4_, and 1.8 mM KH_2_PO_4_). Cells were washed once with 1 mL PBS and then resuspended in 700 μL PBS. To separate cells, suspensions were sonicated twice for 20 s at an amplitude of 3 μm using an MSE Soniprep 150 instrument. A total of 10 μL of sonicated cells was spotted onto a microscope slide and left to dry for 10 min. Once the cells were dry, 5 μL PBS was added to the slide and a cover slip was placed on top. Cells were visualized using an Axioplan 2 microscope (Zeiss) with differential inference contrast (DIC) imaging under ×100 magnification.

To quantify pseudohyphae and yeast cells, the ratio of length to width was determined using Image J (53). A cell was scored as pseudohyphal if its length-to-width ratio was greater than three and it displayed box-shaped ends. Yeast cells had ratios under three and oval-shaped ends. Clear examples of pseudohyphae and yeast were scored visually, whereas intermediate phenotypes (longer but oval-shaped ends) were measured and scored based on the 3:1 length-to-width ratio. The frequencies of pseudohyphae and yeast cells were determined for two experiments, with each group consisting of approximately 150 cells across 8 to 12 fields of view.

### RNA extraction and cDNA synthesis.

For RNA extraction, wild-type and *hst1Δ/Δ* cells were grown on solid media as described above. RNA was extracted from all the cells in a 3-day community (54). Cells were washed once with 500 μL AE buffer (50 mM sodium acetate [pH 5.3] and 10 mM EDTA), resuspended in 500 μL of 1% SDS in AE buffer and 500 μL phenol saturated in citrate (pH 4.3; Sigma; P4682-400ML), and vortexed for 45 min at 4°C with approximately 300 μL of 0.5-mm glass beads (BioSpec Products; 11079105). Nucleic acids were extracted from cell debris by incubating at 65°C for 20 min, freezing on dry ice, and spinning for 10 min at 16,000 × *g* at room temperature. The aqueous layer was removed and extracted twice with 500 μL phenol-chloroform 1:1. RNA was precipitated with 50 μL of 0.3 M sodium acetate in 950 μL ethanol on ice for 15 min. RNA pellets were resuspended in 50 μL nuclease-free water. The RNA concentrations and purity were determined using a Nanodrop instrument.

For cDNA synthesis, 3 μg total RNA was treated with Optizyme DNase according to the product specifications (Fisher; BP81071). Reactions of 30 μL were incubated for 30 min at 37°C. The reaction volume was then increased to 150 μL using nuclease-free water, and the sample was extracted using 200 μL phenol-chloroform. The aqueous phase was transferred to a tube containing 50 μL of 0.3 M sodium acetate in ethanol to precipitate RNA. RNA pellets were resuspended in 30 μL nuclease-free water and analyzed by quantitative PCR (qPCR) to confirm the removal of DNA. cDNA was generated using the iScript-Advanced cDNA synthesis kit (Bio-Rad; 1725038). Reaction mixtures containing ~1 μg DNase-treated RNA were incubated at 42°C for 30 min followed by a 5-min inactivation step at 85°C.

### Gene expression analysis.

Gene expression was analyzed using quantitative PCR (qPCR). cDNA generated from 1 μg DNase-treated RNA was diluted 1:20 in nuclease-free water, and 2 μL diluted cDNA was used in an 8-μL reaction volume containing 200 nM primers and 1× supermix (Bio-Rad; 4364346). Samples were loaded into a 384-well plate and analyzed using a Bio-Rad CFX real-time detection system (1855485). Samples were run for 40 cycles of 10 s at 95°C, 30 s at 55°C, and 1 min at 68°C. A standard curve was prepared from genomic DNA and was used to determine relative starting quantities of each gene in the cDNA samples. Expression was normalized to the *PRI2* gene. Significance was determined using Student’s *t* test using six independent extracts for each condition. Primers are provided in Table 3.

**TABLE 3:** Primers used for RT-qPCR

Gene	Alias	Sequence of:	
		Forward primer	Reverse primer
CPAR2_500980	*PRI2*	CTTGCCCTTGCTCAACC	CCCAATTGTTGACGTCCC
CPAR2_701620	*EFG1*	GCCACAAACGCCTCACAGTC	GGCGTTTGTTGTCCCTGTTG
CPAR2_213640	*NDT80*	CGTACCGCACAACCTCATC	GCAACATCATCATCGGAGC
CPAR2_205990	*BCR1*	CAATGCAAGTGTTGGTGGTGG	GTGCTCGCAGTAACATTAGCG
CPAR2_208600	*CPH1*	CGGTTACCCTGTGCAACAAC	GATGTAGCGCGGCCATTGAC
CPAR2_603440	*CPH2*	GAACGAAGAGGATACTACACAAG	CTTCTTCCAAGTCTTTACAATCGG
CPAR2_800210	*GZF3*	GCAACAGCAGCAGCGAG	GTGTTGGAACCTCTCCTGG
CPAR2_501290	*CZF1*	CCTCATCCGTACCAACAAC	GACGGCATGTGAGACATCCC
CPAR2_805930	*TEC1*	GCCAGAACTTCGATACCCGC	GACCAGCGTCCAAACCTTG
CPAR2_601080	*FLO8*	CTTCGTCAAATGCAACGGGG	GTTGCCACCACCACCAAGAC
CPAR2_803820	*UME6*	CAAATCGACGCCGTTATTGCC	CCGAACCTGACACAAGCCCC
CPAR2_204370	*ACE2*	CGACATTTAAAGGGCCACAG	CCATGCCCTTTACCCCACC
CPAR2_109520	*TUP1*	GCTGCTGGATCTTTAGATCGC	CTATCCAATGACCCTGAAGC
CPAR2_300790	*NRG1*	CCATGGCCTACATGTGATGC	CCCCATAACCAGTATGACCG
CPAR2_803890	*MLS1*	CCAGGAGGTGTCATTACTG	CACCATGAGTGACCCATTGC
CPAR2_106400	*GSC1*	GGCAAGCTCCATTGTTGTGG	GTGCCACTTGGTGTTACCTC
CPAR2_403180	*PGA62*	CCAACCACTCAAACCACCG	GAACAGACTTGGCGGCACCG
CPAR2_803900	*HST1*	CGGAGCTACGGGAGTATAAAG	CTCCAATGTACCGCACCTAC

### Virulence assay.

Virulence assays in Galleria mellonella larvae were performed as described previously (29). Whole spots were harvested and resuspended in 800 μL 1× PBS. Cells were sonicated 20 s for two to three times each at 3 μm using an MSE Soniprep 150 instrument, with additional sonication as needed. The optical density of the suspension was measured, and cells were diluted to an OD_600_ of 4.0 in 1× PBS. Each larva was injected with a 10-μL cell suspension, which is approximately 4.0 × 10^5^ cells. Separately, we counted cells using a hemocytometer and determined that there are approximately equal numbers of cells per OD across all growth conditions.

Prior to injections, Galleria mellonella larvae (waxworms.net, Inc) were weighed and separated into three classes, as follows: small (0.18 to 0.22 g), medium (0.23 to 0.27 g), and large (0.28 to 0.32 g). Equivalent numbers of larvae from each weight class were used for each experimental group. Larvae were incubated at 30°C or 37°C for 24 h before injections. Larvae were injected with 10 μL of 4.0 OD/mL cells in PBS. Larvae injected only with PBS were used as a negative control. Larvae were incubated at 30°C or 37°C for 8 days, and dead larvae were counted and removed every 24 h. Each experimental group consisted of 10 larvae, with duplicate experimental groups for each day of injections performed for 6 separate days. Significance was determined using the log-rank test.
